# Common Dermatologic Disorders in Down Syndrome: Systematic Review

**DOI:** 10.2196/33391

**Published:** 2022-02-08

**Authors:** Megan Lam, Justin Di Lu, Levi Elhadad, Cathryn Sibbald, Raed Alhusayen

**Affiliations:** 1 Michael G. DeGroote School of Medicine McMaster University Hamilton, ON Canada; 2 College of Biological Sciences University of Guelph Guelph, ON Canada; 3 Section of Dermatology Department of Paediatrics SickKids Hospital Toronto, ON Canada; 4 Division of Dermatology Department of Medicine University of Toronto Toronto, ON Canada; 5 Division of Dermatology Sunnybrook Health Sciences Centre Toronto, ON Canada

**Keywords:** autoimmune, comorbidities, trisomy 21, inflammatory, Down syndrome, dermatology, hidradenitis suppurativa, systematic review

## Abstract

**Background:**

Down syndrome (DS) has been associated with cardiovascular, gastrointestinal, and immune-related abnormalities. Several dermatologic conditions, including hidradenitis suppurativa, have also been found to be associated with DS.

**Objective:**

The objective of this study was to characterize the prevalence, presentation, and unique features of dermatologic disorders associated with DS.

**Methods:**

Electronic searches of EMBASE (via Ovid), MEDLINE (via Ovid), and Web of Science databases were conducted on December 14, 2020. Observational studies including case reports of patients with DS presenting with concomitant primary dermatologic disorder were included.

**Results:**

This systematic review captured 40 observational studies and 99 case reports, including 10 observational studies that examined the prevalence of common skin disorders in patients with DS. The most common dermatologic conditions reported includes atopic dermatitis (8 studies, n=180; 19.7% mean prevalence), hidradenitis suppurativa (15, n=478; 3.2%), ichthyosis (4, n=16; 4.7%), lichen nitidus (6, n=6; 1.1%), psoriasis (21, n=65; 4.8%), alopecia areata (27, n=253; 7.4%), vitiligo (8, n=40; 4.4%), onychomycosis (3, n=198; 24.7%), calcinosis cutis (14, n=15), connective tissue nevi (6, n=6), dermatofibroma (3, n=3), melanoma (3, n=3), syringomas (14, n=182; 21.2%), and elastosis perforans serpiginosa (19, n=24; 0.5%).

**Conclusions:**

Our results indicate an increased prevalence of common cutaneous disorders in patients with DS, particularly infectious, inflammatory, autoimmune, and connective tissue conditions. Current guidelines for the screening, general management, and use of systemic immunomodulatory agents in this patient population are lacking. Patients with DS would benefit from screening for dermatologic disorders not otherwise regularly performed for earlier diagnosis and treatment.

**Trial Registration:**

PROSPERO International Prospective Register of Systematic Reviews CRD42021226295; https://www.crd.york.ac.uk/PROSPERO/display_record.php?RecordID=226295

## Introduction

Down syndrome (DS) is one of the most common causes of intellectual disability in high-income countries and has been associated cardiovascular abnormalities, gastrointestinal defects, and immune-related disorders [1]. Dermatologic conditions are also found to be increased in patients with DS, including folliculitis, alopecia areata, and psoriasis [2,3]. A recent survey of 223 families with young adults with DS found that 56% suffered from a dermatological condition [4]. Identification and characterization of associated conditions, particularly those with unique clinical presentations in patients with DS, could help optimize early diagnosis and inform screening.

Thus, the aim of this systematic review was to summarize the prevalence of common dermatologic disorders in patients with DS and to characterize the presentation and unique features of dermatologic disorders when associated with DS.

## Methods

### Overview

This systematic review was conducted in accordance with PRISMA (Preferred Reporting Items for Systematic Reviews and Meta-Analyses) guidelines and was prospectively registered on PROSPERO (International Prospective Register of Systematic Reviews; CRD42021226295). The PRISMA guidelines are an evidence-based guide created to improve the reporting of systematic reviews and follow a 27-item standardized checklist addressing items to include introduction, methods, results, and discussion sections.

### Search Strategy and Inclusion Criteria

We searched EMBASE (via Ovid), MEDLINE (via Ovid), and Web of Science electronic databases from their respective dates of conception to December 14, 2020, with no restrictions. Our search strategy comprised key terms for DS and skin conditions, including specific disorders such as atopic dermatitis, psoriasis, and vitiligo.

We included any observational studies including case reports of patients with DS presenting with concomitant dermatologic disorder including, but not limited to, atopic dermatitis, psoriasis, vitiligo, alopecia areata, acne vulgaris, onychomycosis, hidradenitis suppurativa, and seborrheic dermatitis. Abstracts and unpublished studies were excluded.

### Data Extraction and Synthesis

We screened titles and abstracts (ML and JDL), followed by full texts (ML, LE, and JDL) independently and in duplicate. When necessary, discrepancies were resolved by consulting a senior author (CS and RA). The following data were extracted using a standardized form: study characteristics (author, year, study design, country, and participant source); population characteristics (number of participants, age, sex, race, comorbid conditions, and concurrent medications); disease factors (subtype, age of onset, affected areas, and severity); treatment factors (current treatment, duration, effectiveness, past treatments, and complications of treatment); follow-up interval; and prevalence or incidence statistics if reported.

The quality assessment of included observational studies was performed using the National Institutes of Health’s National Heart Lung and Blood Institute quality assessment tools. The National Institutes of Health quality assessment tools have been used in the systematic evidence review of national updates to clinical guidelines and offer nonnumeric methods for critical appraisal of the internal validity of a study, with specific tools for individual types of study designs, including controlled intervention, cross-sectional, and case-control studies. Reviewers respond “yes,” “no,” or “cannot determine/not reported/not applicable” in response to each item in the tool, which includes sources of bias, confounding, study power, and strength of causality, to assess the risk of bias in the study and determine a rating of “good,” “fair,” or “poor” quality. Case reports were evaluated for methodological quality using an updated 8-item tool proposed by Murad et al [5]. We anticipated that much of the body of evidence from this systematic review would consist primarily of uncontrolled clinical observations, and this tool was selected as it provided a tailored approach to the assessment of evidence derived from case reports and case series, based on 4 domains (selection, ascertainment, causality, and reporting).

Qualitative syntheses for study characteristics, as well as key characteristic, outcomes, and treatment regimens, were summarized for each dermatologic condition. Where applicable, weighted means were calculated for observational studies reporting the prevalence of skin disorders in persons with DS.

## Results

### Overview

**Figure 1:**
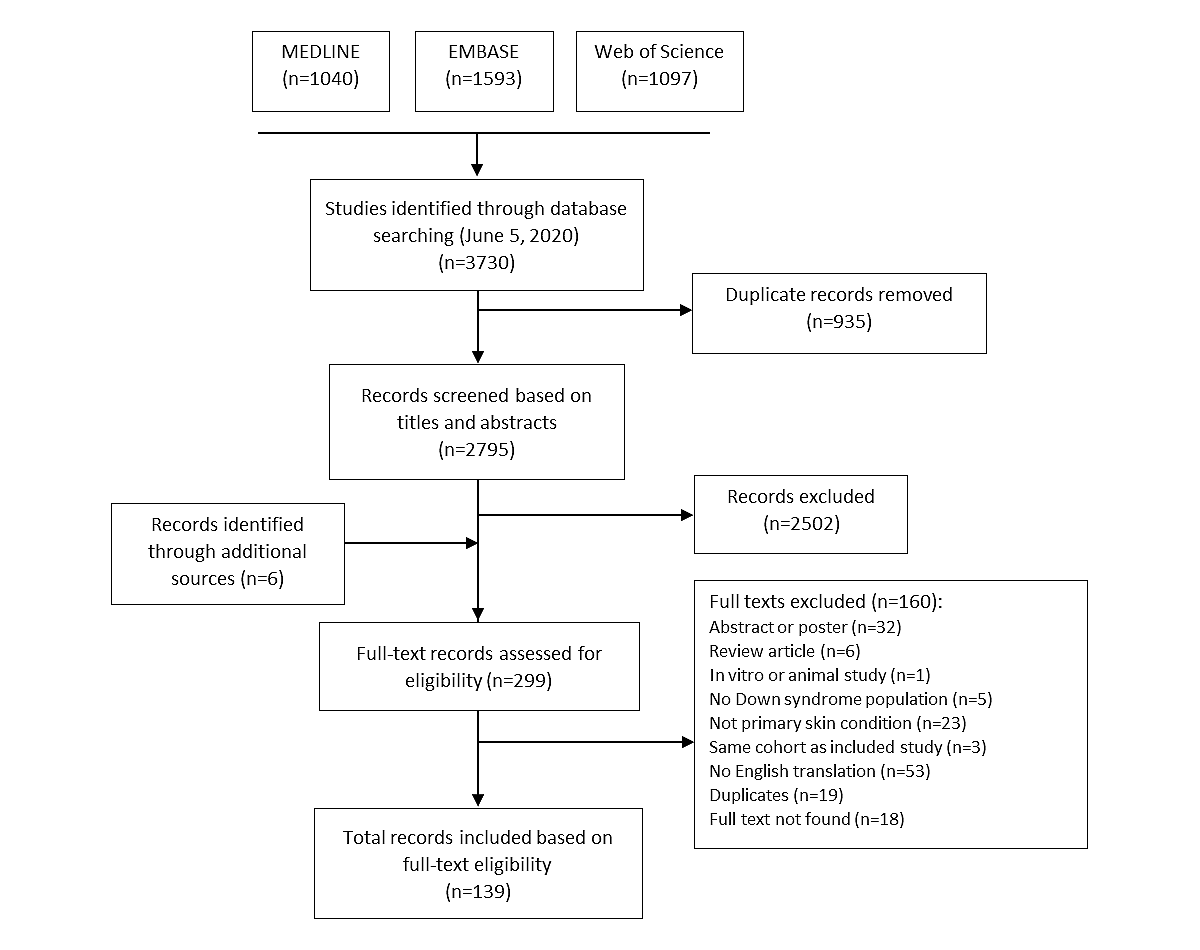
Study selection methodology.

Ultimately, 40 observational studies and 99 case reports were included in this systematic review (Table 1 and Figure 1).

**Table 1:** Summary of search results by dermatologic condition.

Dermatologic condition		Number of studies				Weighted mean prevalence,^a^ % (n/N)
		Case report, n	CS/Cohort,^b^ n	Observational, n		
**Inflammatory skin conditions**						
	Acne vulgaris	0	0	7	14.7 (149/1017)	
	Atopic dermatitis	2	0	6	19.7 (178/903)	
	Cheilitis	0	0	6	8.4 (68/805)	
	Folliculitis	1	0	7	21.2 (213/1006)	
	Hidradenitis suppurativa	2	1	6	3.2 (425/13266)	
	Ichthyosis	2	0	2	4.7 (14/298)	
	Keratosis pilaris	0	0	9	8.6 (97/1134)	
	Lichen nitidus	5	0	1	1.1 (—^c^)	
	Pityriasis rubra pilaris	3	0	0	—	
	Psoriasis	14	1	6	4.8 (46/953)	
	Seborrheic dermatitis	0	0	8	18.5 (212/1149)	
**Autoimmune skin conditions**						
	Alopecia areata	11	5	11	7.4 (190/2574)	
	Vitiligo	3	0	5	4.4 (31/709)	
**Infectious skin conditions**						
	Leishmaniasis	4	0	0	—	
	Onychomycosis	0	2	3	24.7 (188/761)	
	Scabies	7	0	—	—	
	Tinea capitis	0	0	1	2.5 (6/243)	
	Tinea corporis	0	0	2	2.0 (9/446)	
	Tinea cruris	0	0	1	8.4 (18/214)	
	Tinea pedis	0	0	4	30.9 (190/615)	
**Cutaneous birthmarks, tumors, and depositions**						
	Café au lait macules	0	0	5	3.8 (24/633)	
	Calcinosis cutis	13	1	1	3.0 (—)	
	Connective tissue nevi	6	0	0	—	
	Dermatofibroma	3	0	0	—	
	Melanoma	3	0	0	—	
	Syringoma	8	0	6	21.2 (174/821)	
**Other skin conditions**						
	Acanthosis nigricans	0	0	3	30.7 (67/218)	
	Cutis marmorata	0	0	3	8.4 (28/335)	
	EPS^d^	16	2	1	0.5 (1/203)	
	Other case reports^e^	7	—	—	—	

^a^Weighted mean prevalence of patients with dermatologic condition in a population with Down syndrome, calculated from values reported in observational studies.

^b^CS/Cohort: Case series or cohort studies with no prevalence value provided.

^c^Not available.

^d^EPS: elastosis perforans serpiginosa.

^e^Other case reports examined patients with actinomycetoma, cheilitis granulomatosa, epidermolysis bullosa, generalized perforating granuloma annulare, keratosis follicularis spinulosa decalvans, reactive perforating collagenosis, and familial urticaria pigmentosa.

Ten of the observational studies reported the prevalence of cutaneous disorders in general in populations with DS (Table 2).

Case reports were primarily carried out in the United States (n=28), Japan (n=13), and Italy (n=11). Quality assessment yielded the following ratings for case reports: good, n=25; fair, n=70; and poor, n=5. It also yielded the following ratings for observational studies: good, n=25; fair, n=12; and poor, n=3.

**Table 2:** Observational studies examining prevalence of dermatologic conditions in patients with Down syndrome.

Study	Country	Study setting	Criteria for dermatologic diagnosis	n^a^	Mean age (years), (range)	M/F^b^	Comorbidities	RoB^c^
Camacho et al, 2014 [6]^d^	Spain	Trichology unit of the Department of Dermatology of the Virgen Macarena University Hospital; Jan 2001-Jan 2011	Focused clinical exam	15	11.2 (7-16)	8/7	Hypothyroidism (n=6); celiac disease (n=6); epilepsy (n=1)	Good
Camacho et al, 2014 [6]	Spain	Special Education Schools in Seville; March 1, 2011-April 30, 2011	Focused clinical exam	57	16.7 (2-29)	34/23	Hypothyroidism (n=22); celiac disease (n=28)	Good
Carter, 1976 [7]	United States	Southbury Training School	Focused clinical exam by investigators, with ancillary testing when necessary	214	—^e^ (12-48)	128/86	3 of the 4 patients with vitiligo had AA^f^	Fair
Daneshpazhooh et al, 2007 [8]	Iran	Schools for children with special educational needs and centers in the Karaj and Sharyar provinces in Tehran, Iran, 2002	—	100	11.2 (3-20)	47/53	—	Good
Ercis et al, 1996 [9]	Turkey	Hacettepe University Children’s Hospital Clinical Genetics Department; June 1991-Sept 1992	Focused clinical exam by an expert dermatologist	71	2.8 (0-25)	41/30	—	Good
Firsowicz et al, 2019 [10]	United States	Children with DS^g^ with ICD^h^-10 code Q90.0 at Texas Children’s Hospital Dermatology Clinic; May 2001-August 2018	Retrospective chart review	243	—	—	—	Good
Gunes Bilgili, 2011	Turkey	Outpatient pediatric and dermatology clinic	Focused clinical exam	50	2.2 (0-11)	28/22	—	Good
Rork et al, 2020 [11]	United States	At least 1 outpatient dermatology visit from Jan 1, 2008, to April 1, 2018, with ICD-9/ICD-10 codes 758.0/Q90.0 (DS or trisomy 21)	Retrospective chart review	101	19.7 (0-66)	62/39	Hypothyroidism (2 out of 7 AA patients)	Good
Schepis et al, 2002 [2]	Italy	Oasi Institute for Research on Mental Retardation and Brain Aging, consecutively seen 1990-2000	Focused clinical exam, with ancillary testing where applicable	203	11.7 (—)	125/78	Hypothyroidism (n=40)	Good
Sureshbabu et al, 2011 [12]	India	Consecutive DS patients recruited from special schools or homes in and around Pondicherry	Focused clinical exam by both a pediatrician and a dermatologist	95	12.0 (0-40)	59/36	—	Good
Tenenbaum et al, 2012 [13]	Israel	Adults with DS hospitalized at the Hadassah Medical Centers; 1988-2007	Retrospective chart review	120	36.3 (18-73)	73/47	—	Good

^a^Total number of patients with Down syndrome.

^b^M/F: male/female.

^c^RoB: risk of bias.

^d^Camacho et al [6] had 2 separate cohorts of patents with Down syndrome.

^e^Not available.

^f^AA: alopecia areata.

^g^DS: Down syndrome.

^h^ICD: International Classification of Diseases and Related Health Problems.

### Inflammatory Skin Conditions

#### Atopic Dermatitis

Six observational studies reported the prevalence of atopic dermatitis (AD) in their cohorts with DS. The mean prevalence was 19.7% (178 patients with AD out of 903 total patients with DS) [2,6,7,9,10,14]. The study by Schepis et al [14] in 1997 was the only observational study to examine AD specifically and compared its prevalence in a group with DS to a control group. The DS and control groups were reported to have the same prevalence of AD (3.0%).

Two case reports of patients with DS having scabies were also reported to have a history of AD [15,16].

#### Hidradenitis Suppurativa

Six observational studies with a mean prevalence of 3.2% (425/13266) of hidradenitis suppurativa (HS) in patients with DS were included [6,10,11,17-19]. One study reported a significantly increased risk of HS in patients with DS compared with controls after adjusting for age, sex, race, and obesity (odds ratio 5.24, 95% CI 4.62-5.94) [18]. Six other observational studies reported a weighted mean prevalence of 2.5% (40/1609) of DS among patients with HS [20-25]. The mean age of onset for HS in patients with DS in observational studies was 14.3 years.

There were also 2 case reports and 1 case series examining HS in patients with DS [26-28].

#### Ichthyosis

Two observational studies reported the prevalence of ichthyosis vulgaris in patients with DS, with a mean prevalence of 4.7% (14/298) [2,12].

Two case reports included patients with features of ichthyosis vulgaris; both cases were reported to clinically resemble ichthyosis vulgaris and were supported by histologic findings but were missing features of early onset in life and positive family history [29,30].

#### Lichen Nitidus

One observational study reported a prevalence of 1.1% (1/95) of lichen nitidus (LN) in patients with DS [12].

Five case reports of LN were reported (Multimedia Appendix 1) [31-35]. One other case report in French (not included in this systematic review) presented a patient with DS having LN with associated megacolon [36].

#### Pityriasis Rubra Pilaris

Three case reports of pityriasis rubra pilaris (PRP) were found (Multimedia Appendix 2) on 2 female patients with circumscribed juvenile PRP (type IV) [37,38] and 1 male patient with classic juvenile PRP (type III) [39]. Accordingly, 2 patients were treated with oral etretinate with long-term control of symptoms [38,39], while 1 patient was treated effectively with topical 0.1% trans retinoic acid [37].

#### Psoriasis

Six observational studies reported the prevalence of psoriasis in a population with DS, with a weighted mean prevalence of 4.8% (46/953) [2,6,7,10,11,13]. One observational study reported 2 (0.4%) patients with DS in a cohort of 419 children with psoriasis [40].

Moreover, there were 14 case reports and 1 case series with 17 patients in total, where 3 (17.6%) of the patients had psoriatic arthritis (Multimedia Appendix 3) [29,41-54]. Six studies reported failed or ineffective systemic treatment with immunosuppressants [41,45,46,51,52], including the study by Adamcyzk et al [41], who reported discontinuing cyclosporin A treatment due to elevated liver enzymes, and Alcaide et al [42], who reported contraindications for cyclosporin and methotrexate due to renal and liver problems, respectively. Of the 8 patients treated successfully with systemic immunosuppressive treatments, 5 patients were treated with biologics (etanercept [41,42], ustekinumab [52], infliximab [51], adalimumab [46]), and 3 with conventional systemic medications including cyclosporin [47], azathioprine [45], and oral or intramuscular hydrocortisone [53].

### Autoimmune Skin Conditions

#### Alopecia Areata

Eleven observational studies examined the prevalence of alopecia areata (AA) in populations with DS, with a weighted mean prevalence of 7.4% (190 patients with AA, out of 2574 patients with DS), and a range of 1.4%-21.0% [2,6-12,55-57]. One observational study reported 5 (1.3%) patients with DS in a cohort of 392 patients with AA [58].

Three observational studies examined only patients with both AA and DS, with a total of 44 patients and a weighted mean age of onset of 7.0 years (Multimedia Appendix 4) [59-61]. Lima Estafan et al [59] also reported a mean duration of 2.7 years and recurrence in 27.7% of patients. The study found no concomitant vitiligo or autoimmune disease, as well as no first-degree relatives with AA [59]. By contrast, Ramot et al [60] reported that 8 (57%) of patients had a 1st or 2nd degree relative with AA. Ramot et al [60] and Schepis et al [61] reported 6 (42.9%) and 4 (33.3%) with thyroid abnormalities, and 1 (7.1%) and 4 (33.3%) with celiac disease.

In addition, 11 case reports and 2 case series presented 14 patients with AA and DS, with a mean age of onset of 7.0 (SD 4.5) (Multimedia Appendix 5) [26,49,54,62-71]. Three studies presented patients with normal hair growth in areas of comorbid inflammatory skin disease (HS [26] and psoriasis [49,54]), also known as the Renbok phenomenon. Moreover, 5 patients had concomitant hypothyroidism [26,49,67,69,71], with 1 patient demonstrating complete resolution of hair regrowth 12 months after starting thyroxine treatment [69].

#### Vitiligo

Five observational studies with a weighted mean prevalence of 4.4% (31/709) of vitiligo in patients with DS were included [6-8,10,12]. Two observational studies reported a mean prevalence of 0.6% (6/1030) of DS in a cohort of patients with vitiligo [72,73].

Three case reports on patients with DS having vitiligo were included, associated with LN (aged 4 years, female) [31], leishmaniasis (aged 35 years, male) [74], and PRP (aged 30 years, female) [37]. One patient also had hypothyroidism and type II diabetes mellitus [74].

### Infectious Skin Conditions

#### Fungal Infections

Three observational studies examining the prevalence of onychomycosis among patients with DS had a weighted mean prevalence of 24.7% (188/761) [2,7,10,11]. Two other observational studies examining the prevalence of DS in patients with onychomycosis had a mean prevalence of 30.3% (10/33) [75,76]. One other cohort study examining only patients with DS having onychomycosis treated with terbinafine reported that all 32 patients had negative cultures after 24 weeks of treatment [77].

Additionally, 4 observational studies reported a mean weighted prevalence of 30.9% (190/615) of tinea pedis; 2 studies reported a weighted mean prevalence of 2.0% (9/446) of tinea corporis; 1 study reported a prevalence of 8.4% (18/214) of tinea cruris; and 1 study reported a prevalence of 2.5% (6/243) of tinea capitis.

Goulen et al [78] reported the successful treatment of a 5-year-old female patient with a Trichophyton rubrum-infected toenail, with 12 months of griseofulvin, followed by 6 months of daily terbinafine.

#### Other Infections

There was 1 observational study of a scabies outbreak among persons with mental disability, which reported an index case of a 16-year-old patient with DS [79]. There were also 7 case reports of scabies (Multimedia Appendix 6) [15,16,80-84], where 4 of the cases reported an initial misdiagnosis of scabies, and the patients were instead treated ineffectively for presumed onychomycosis, psoriasis, eczema, tinea corporis, and psoriasiform dermatitis [16,80-82,84]. There were also 4 case reports of leishmaniasis (Multimedia Appendix 7) [74,85-87] and 1 case report of actinomycetoma [88].

### Cutaneous Birthmarks, Tumors, and Depositions

#### Calcinosis Cutis

Thirteen case reports and 1 case series reported 15 patients with calcinosis cutis, where 12 were diagnosed with milia-like calcinosis cutis [89-100], 1 with dystrophic calcinosis cutis [101], and 1 unspecified case (Multimedia Appendix 8) [102]. There were no reports of abnormal laboratory values, including serum calcium, phosphate, and parathyroid hormone levels. Six studies reported concomitant presentation of syringomas, with 5 cases of palpebral syringomas [90,94,96,100,102], and 3 studies that reported perilesional syringomas [90,97,102].

#### Connective Tissue Nevi

Six case reports presenting patients with DS having collagenomas or connective tissue nevi were included, with a mean age of 22.8 (SD 14.9) years [30,95,103-106]. No history of trauma was reported.

#### Dermatofibroma

Three cases of multiple dermatofibromas were included (Multimedia Appendix 9) [107-109], commonly defined as the development of 5 to 8 lesions within 4 months. The number of lesions at the time of report ranged from 6 to approximately 30. None had evidence of immunosuppression, although 1 patient presented with mild lymphopenia [109], and another with a history of acute megakaryoblastic leukemia [107].

One other case report in Spanish (not included in this systematic review) presented 3 patients with DS having multiple dermatofibromas, where 1 patient was immunosuppressed receiving methotrexate [110].

#### Melanoma

Three patients with cutaneous melanomas were reported (Multimedia Appendix 10) [111-113]. Jafarian et al [111] reported an 11-year-old patient with a stage IIA melanoma of the leg. Satge et al [112] reported a 19-year-old female patient with superficial spreading melanoma (Clark level II) in the lumber region. Lastly, Nakano et al [113] reported a 39-year-old patient with an acral lentiginous melanoma (Clark level V) of the right foot with central ulcer. No evidence of metastasis was found in any of the patients at the time of presentation, and all were treated with surgical excision.

#### Syringomas

Six observational studies examined the prevalence of syringomas in patients with DS, with a weighted mean prevalence of 21.2% (174/821) (Multimedia Appendix 11) [2,6-8,114,115]. Two of these observational studies only investigated for syringomas, published in 1964 and 1991 [114,115]. Feingold et al [115] also included an age-matched control group, which had a prevalence of 2.0% of syringomas, and reported that cases of syringomas in patients with DS did not present concurrent hypothyroidism or congenital heart disease.

Eight case reports included patients with DS having syringomas [90,94,96,100,102,104,116,117]. Five reported periorbital or palpebral syringomas [90,96,100,102,117]. One report described a case of eruptive syringomas over the trunk over the course of 1 month [116].

### Other Skin Conditions

#### Elastosis Perforans Serpiginosa

One observational study reported a prevalence of elastosis perforans serpiginosa (EPS) in 203 patients with DS of 0.5% [2].

Moreover, 16 case reports and 2 case series examined 23 patients with EPS, with a mean age of 22.1 (SD 9.2) years (Multimedia Appendix 12) [83,118-134]. Three studies reported spontaneous resolution of lesions, ranging from 6 months to 3 years [129,133,134]. Topical steroids were reported to be ineffective in 7 cases [83,118,122,123,132,133].

#### Other Case Reports

Other case reports involving primary skin conditions in patients with DS include anetoderma secondary to folliculitis [135], cheilitis granulomatosa [136], epidermolysis bullosa [137], generalized perforating granuloma annulare [138], keratosis follicularis spinulosa decalvans [139], reactive perforating collagenosis [140], and familial urticaria pigmentosa [141].

## Discussion

### Principal Findings

This systematic review captured 40 observational studies and 99 case reports, including 10 observational studies that examined the prevalence of common skin disorders in general in patients with DS. Our results indicate a potential association between DS and common cutaneous disorders including alopecia areata, acne vulgaris, hidradenitis suppurativa, and seborrheic dermatitis, although the scope of evidence in the literature is quite limited. Less common skin disorders including calcinosis cutis, eruptive syringomas, and multiple dermatofibromas were frequently described in case reports of patients with DS. Connective tissue conditions were also observed frequently in patients with DS including EPS, collagenomas, and reactive perforating collagenosis. Some cases of EPS also had high incidence of joint hyperextensibility and premature skin aging [120,126], suggesting a presence of connective tissue dysplasia.

Autoimmune conditions including psoriasis and AA have been linked to immune dysregulation in patients with DS [26,50]. Increased activity of CD4 T-lymphocytes and their proinflammatory cytokines (IFN-γ [interferon gamma] and TNF-α [tumor necrosis factor alpha]) are also involved in psoriasis pathogenesis [46]. Patients with DS may also therefore be more prone to severe cases of infestation and bacterial proliferation in the skin [10,86]. The cases of scabies reported in this review were extensive, tended to be generalized to the whole body, and were often clinically misdiagnosed and treated ineffectively, for instance as AD or psoriasis, before the diagnosis of scabies was made. The most recent guidelines set by the American Academy of Pediatrics for the management of children with DS do not provide any skin care recommendations for patients with DS [142]. Given the prevalence of skin disorders as outlined in this review, patients with DS would benefit from screening of dermatologic disorders that are not otherwise regularly performed for earlier diagnosis and treatment. However, patients with DS may experience difficulties accessing adequate services for the screening and treatment of cutaneous disease, for instance, given cognitive disabilities, social barriers, and potentially impairing comorbid physical and mental health conditions. Potential difficulties adhering to screening and treatment regimens, as well as preventative measures such as sun protection, may also pose challenges to interventions.

With the exception of 1 case [82], none of the patients were medically immunosuppressed. Nevertheless, most reports of scabies included in this review had superimposed bacterial infections and received antibiotic treatment. Similarly, with infectious and inflammatory conditions in and around the pilosebaceous unit including acne vulgaris, folliculitis, and HS, immunodeficiency predisposes patients to these conditions. An association with HS and DS has been previously outlined in a recent meta-analysis by Lam et al [143], which not only demonstrated a significant association, but also a younger age of onset for patients with DS for HS.

Standardized guidelines for systemic immunomodulatory agents in this patient population are lacking, and reports of systemic immunosuppressants in the treatment of cutaneous disorders in patients with DS are limited. The theoretical increased risk of infection and other complications, possibly due to concerns of low compliance or other comorbidities including congenital heart, haemato-oncological and endocrinological disorders, as well as immunological alterations lead to prescriber hesitation when considering biologics in severe cases refractory to other treatments [52]. Several patients described in this review presented cases where treatment with immunomodulatory agents were discontinued due to adverse effects or contraindicated due to preexisting conditions; however, considerations in the safety of these systemic agents in patients with DS remain unclear [52,144].

### Limitations

Our study had several limitations. First, our calculated prevalence of skin conditions may have overestimated real prevalence, as studies that either did not assess for or found no cases were not included in weighted mean calculations. Our conclusions based on prevalence are also limited by insufficient studies with age-matched controls to provide comparison of prevalence in a matched population. Selection bias for patients included in case reports and case series limits interpretation. Additionally, patients with DS may be more likely to interact with health care providers given their increased risk of comorbidities and medical complications, which may result in an increase in diagnoses of cutaneous disease, among other diseases. Lastly, 53 studies were not included due to language restrictions.

### Conclusions

This review highlights the need for additional data on the true prevalence and onset of dermatologic conditions in persons with DS. Particularly for conditions including psoriasis and HS, early diagnosis and treatment as well as appropriate screening will be important. Patients with DS may also be at an increased risk of cutaneous infections, and possible misdiagnoses could lead to increased severity at presentation. For patients with DS who may have difficulty communicating their symptoms, screening for and recognizing the associated skin disorders in this population should be incorporated as a necessary part of care.

## Appendix

Multimedia Appendix 1 Summary of case reports of Down syndrome patients with lichen nitidus.

Multimedia Appendix 2 Summary of case reports of Down syndrome patients with pityriasis rubra pilaris.

Multimedia Appendix 3 Summary of case reports of Down syndrome patients with psoriasis.

Multimedia Appendix 4 Observational studies examining only patients with both Down syndrome and alopecia areata.

Multimedia Appendix 5 Summary of case reports of Down syndrome patients with alopecia areata.

Multimedia Appendix 6 Summary of case reports of Down syndrome patients with scabies infestation.

Multimedia Appendix 7 Summary of case reports of Down syndrome patients with leishmaniasis infestation.

Multimedia Appendix 8 Summary of case reports of Down syndrome patients with calcinosis cutis.

Multimedia Appendix 9 Summary of case reports of Down syndrome patients with dermatofibromas.

Multimedia Appendix 10 Summary of cases of Down syndrome patients with confirmed melanoma.

Multimedia Appendix 11 Summary of case reports of Down syndrome patients with syringoma(s).

Multimedia Appendix 12 Summary of case reports of Down syndrome patients with elastosis perforans serpiginosa.

## Abbreviations

AA: alopecia areata
AD: atopic dermatitis
DS: Down syndrome
EPS: elastosis perforans serpiginosa
HS: hidradenitis suppurativa
IFN-γ: interferon gamma
LN: lichen nitidus
PRISMA: Preferred Reporting Items for Systematic Reviews and Meta-Analyses
PROSPERO: International Prospective Register of Systematic Reviews
PRP: pityriasis rubra pilaris
TNF-α: tumor necrosis factor alpha
