# Supplementary material for: Common Dermatologic Disorders in Down Syndrome: Systematic Review
Source: JMIR Dermatol. 2022 Feb 8;5(1):e33391. doi: 10.2196/33391 (PMC10334906; doi:10.2196/33391)
Supplement: Multimedia Appendix 1 [file derma_v5i1e33391_app1.docx]

# Summary of case reports of Down syndrome patients with lichen nitidus

| **Study** | **Country** | **Age, Sex** | **Onset** | **Affected areas** | **Comorbidities** | **Current treatment** | **Past treatment** | **ROB** |
| --- | --- | --- | --- | --- | --- | --- | --- | --- |
| *Laxmisha, 2006* | India | 2, F | 3 months prior | Abdomen, trunk, face, arms, legs, genitalia | NR | NR | Oral cetirizine 2.5 mg/day, with no response to treatment after 1 month | Good |
| *Henry, 2009* | USA | 3, M | 1 year prior | Face, abdomen | NR | Observation | Low-potency topical corticosteroids, topical immunomodulators, ammonium lactate lotion with no improvement | Fair |
| *Agarwal, 2009* | India | 4, F | 1 month prior | Bilateral legs | Segmental vitiligo | NR | NR | Fair |
| *Botelho, 2012* | Brazil | 4, F | 3 years prior | Face, trunk, upper and lower limbs, genitalia | Hypothyroidism | Moisturizing cream with improvement at 2 months | NR | Good |
| *Guliani, 2019* | India | Pre-school, M | 1 year prior | Whole body (no lesions on scalp, mouth, palms, soles, or nails) | NR | Topical steroids and moisturizers | NR | Fair |

**Abbreviations:** CR – complete resolution; NR – not reported; ROB – risk of bias assessment
