# Supplementary material for: Common Dermatologic Disorders in Down Syndrome: Systematic Review
Source: JMIR Dermatol. 2022 Feb 8;5(1):e33391. doi: 10.2196/33391 (PMC10334906; doi:10.2196/33391)
Supplement: Multimedia Appendix 2 [file derma_v5i1e33391_app2.docx]

# Summary of case reports of Down syndrome patients with pityriasis rubra pilaris (PRP)

| **Study** | **Country** | **Age, Sex** | **Type** | **Age of onset** | **Affected areas** | **Other skin conditions** | **Effective treatment** | **Failed or previous treatments** | **ROB** |
| --- | --- | --- | --- | --- | --- | --- | --- | --- | --- |
| *Terasaki, 2004* | Japan | 15, M | Classic juvenile PRP (type III) | 15 | Flexor aspect of limbs, trunk | NR | Etretinate 0.15 mg/kg/day; well controlled | Failed: Vitamin A, topical urea ointment, tacalcitol ointment; PUVA, topical maxacalcitol | Good |
| *Holden, 1989* | UK | 23, F | Circumscribed juvenile PRP (type IV) | 18 | Knees, elbows, trunk | Palmoplantar keratoderma | Etretinate; well controlled | NR | Poor |
| *Hazini, 1988* | Italy | 30, F | Circumscribed juvenile PRP (type IV) | 6 | Knees, elbows, abdomen | Vitiligo | topical 0.1% trans-retinoic acid with improvement of follicular hyperkeratosis | NR | Good |

**Abbreviations:** NR – not reported; PUVA – psoralen and ultraviolet A; ROB – risk of bias assessment
