# Supplementary material for: Common Dermatologic Disorders in Down Syndrome: Systematic Review
Source: JMIR Dermatol. 2022 Feb 8;5(1):e33391. doi: 10.2196/33391 (PMC10334906; doi:10.2196/33391)
Supplement: Multimedia Appendix 3 [file derma_v5i1e33391_app3.docx]

# Summary of case reports of Down syndrome patients with psoriasis

| **Study** | **Country** | **Age, Sex** | **Age of onset** | **Subtype** | **Comorbidities** | **Effective treatment** | **Failed or previous treatments** | **ROB** |
| --- | --- | --- | --- | --- | --- | --- | --- | --- |
| *Tudor, 1976* | USA | 5, M |  | NR | Arthritis | Hydrocortisone (oral and intramuscular injections), with Cordran tape and Betamethasone  cream, with partial remission | NR | Poor |
| *Morita, 2019* | Japan | 8, F | 8 months prior | Annular pustular (PASI 31.8) | GH-deficient dwarfism (GH replacement) | Cyclosporin (5 mg/kg/d) with CR | NR | Good |
| *Rotchford, 1961* | USA | 9, F | 18 months prior | NR | NR | None | 3x-irradiations, tar baths, P&S liquid to the scalp, mercury ointments | Poor |
| *Adamcyzk, 2017* | Poland | 12, F | 4 | Plaque (PASI 41) | NR | 24-week courses of etanercept as needed | Oral cyclosporine A (3.5 mg/kd/day) tapered down due to elevated liver enzymes and completely withdrawn due to multiple viral warts | Good |
| *Marmon, 2012* | USA | 12, M | 9 | Chronic plaque (PASI 1) | Asthma, GERD | Adalimumab 40 mg injections every other week, significant improvement at 4 weeks, and well controlled for 2 years | NR | Fair |
|  |  | 16, M | 6 | Chronic plaque | Diabetes, hypothyroidism | Topical calcipotriene and tacrolimus 0.1% ointment with significant improvement | NR |  |
|  |  | 20, M | 15 | Chronic plaque (PASI 4) | Tetralogy of Fallot | Methotrexate (15 mg) weekly and Adalimumab (40mg) every other week with significantly sustained improvement | Methotrexate (15 mg) and Etanercept (50 mg) weekly |  |
| *Sugiura, 2015* | Japan | 15, M | 2 | NR | Arthritis | 3 mg/kg infliximab every 2 months, with CR | NR | Fair |
| *Hedayati, 2020* | USA | 17, F | 15 | Annular pustular | NR | Augmented betamethasone dipropionate 0.05% ointment with significant improvement in 1 month | Mid and high potency topical steroids | Fair |
| *Wylie, 2011* | UK | 20, M | NR | Plaque | Alopecia Areata | NR | NR | Fair |
| *Jorgensen, 1995* | France | 23, M | NR | NR | Arthritis | Methylprednisone and azathioprine, with CR | Acitretin 25 mg/day with partial improvement | Fair |
| *Nomura, 1999* | Japan | 24, M | 10 | NR | Ichthyosis | NR | NR | Fair |
| *Alcaide, 2008* | Spain | 30, M | NR | PASI 14 | Hepatitis C infection | Etanercept (25 mg injections twice weekly), with dramatic improvement in 1 month to PASI 4 | Topical agents; immunosuppressant drugs (cyclosporin, methotrexate) contraindicated due to renal and liver problems | Fair |
| *Schepis, 2017* | Italy | 30, M | 28 | NR | Hypothyroidism; Alopecia areata | NR | NR | Poor |
| *Talamonti, 2012* | Italy | 31, M | 14 | Plaque (PASI 12) | NR | Ustekinumab 45 mg injections, with significant improvement to PASI 3 and 0.8 after 16 and 52 weeks respectively | Topical medications (tar, calcipotriol, corticosteroids), systemic therapies (cyclosporine and methotrexate) | Fair |
| *Sismour, 2019* | USA | 45, F | NR | NR | Atrioventricular septal defect, secondary polycythemia, hyperlipidemia | None | 0.025% triamcinolone acetonide cream twice daily | Fair |
| *Fargnoli, 2004* | Italy | 56, M | 25 | NR | Hodgkin’s lymphoma; Kaposi’s sarcoma | None | Topical corticosteroids and vitamin D derivatives; recombinant interferon α-2a injections with worsening of psoriatic plaques | Good |

**Abbreviations**: CR – complete resolution; GERD – gastroesophageal reflux disease; GH – growth hormone; NR – not reported; PASI – psoriasis area severity index; ROB – risk of bias assessment
