# Supplementary material for: Common Dermatologic Disorders in Down Syndrome: Systematic Review
Source: JMIR Dermatol. 2022 Feb 8;5(1):e33391. doi: 10.2196/33391 (PMC10334906; doi:10.2196/33391)
Supplement: Multimedia Appendix 4 [file derma_v5i1e33391_app4.docx]

# Observational studies examining only patients with both Down syndrome and alopecia areata

| **Study** | **Country** | ***n*^a^ (% female)** | **Mean age of onset (years)** | **% with patch-type AA** | **Family history** | **Comorbidities** | **ROB** |
| --- | --- | --- | --- | --- | --- | --- | --- |
| *Lima Estafan, 2013* | Brazil | 18 (39%) | 2.7 | 83.4% | No 1^st^ degree relative with AA | 7 with atopy (asthma, rhinitis); none with vitiligo or autoimmune disease | Good |
| *Ramot, 2013* | Israel | 14 (NR) | 6.8 | 79% | 8 (57%) patients with 1^st^ or 2^nd^ degree relatives with AA | 6 with thyroid abnormalities, 1 with celiac disease | Fair |
| *Schepis, 2005* | Italy | 12 (8%) | 13.7 | 100% | NR | 4 with hypothyroidism, 4 with celiac disease | Fair |

^a^*n* signifies number of patients with both Down syndrome and alopecia areata

**Abbreviations**: AA – Alopecia areata; NR – not reported; ROB – risk of bias assessment
