# Supplementary material for: Common Dermatologic Disorders in Down Syndrome: Systematic Review
Source: JMIR Dermatol. 2022 Feb 8;5(1):e33391. doi: 10.2196/33391 (PMC10334906; doi:10.2196/33391)
Supplement: Multimedia Appendix 5 [file derma_v5i1e33391_app5.docx]

# Summary of case reports of Down syndrome patients with alopecia areata

| **Study** | **Country** | **Age, Sex** | **Onset** | **Subtype** | **Comorbidities** | **Effective treatment** | **Failed or previous treatments** | **ROB** |
| --- | --- | --- | --- | --- | --- | --- | --- | --- |
| *Bimbi, 2020* | Greece | 6, M | 1 month prior | AA | NR | Tacrolimus 0.1% under occlusion, CR in 6 months | Methylprednisolone aceponate 0.1% cream and 5% minoxidil lotion b.i.d.; tacrolimus ointment; oral steroid therapy (stopped due to adverse effects) | Fair |
| *Dourmishev, 2000* | Bulgaria | 8, M | 6 months prior | AA | NR | Anthralin 0.1%-0.5% cream; significant improvement within 2 months | NR | Good |
| *Sethuraman, 2006* | India | 8, M | 4 y/o | AU | NR | Oral betamethasone (3 mg) and minoxidil 2% lotion; significant improvement within 3 months | Topical corticosteroids and minoxidil | Fair |
| *Bordel-Gomez, 2008* | Spain | 9, M | Since birth | Congenital triangular alopecia | NR | NR | Topical corticosteroids | Fair |
| *Storm, 2000* | Germany | 9, F | 3 years prior | AA | Celiac disease, congenital hypothyroidism | Gluten free diet; CR after 8 months | Zinc tablets with normal growth after 18 months, but recurrence 6 months after therapy ceased | Fair |
| *Pirgon, 2009* | Turkey | 10, F | 6 months prior | AA | Diabetic ketoacidosis; hypothyroidism | NR | NR | Fair |
| *Hatamochi, 1984* | Japan | 13, F | 8 y/o | AU | NR | Topical 0.3% DNCB; hair regrowth in 1 month | PUVA; 1.5 mg q.d. dexamethasone, with excellent regrowth of scalp hair, recurrence upon tapering of dosage | Fair |
| *Rachubinski, 2019* | USA | 15, M | 13 y/o | AA | NR | Tofacitinib 5 mg bid + topical clobetasol external solution (0.05%) q.d., CR after 2 months | Intralesional steroid injections (slight and transient localized hair regrowth) | Fair |
|  |  | 16, F | 6 y/o | AA | NR | Tofacitinib 5 mg b.i.d., with CR after 3 months | Fluocinonide in FAPG cream; triamcinolone acetonide injectable suspension |  |
| *Norton, 1999* | USA | 16, M | From birth | AU | NR | NR | NR | Fair |
| *Wylie, 2011* | UK | 20, M | NR | AA | NR | NR | NR | Fair |
| *Molinelli, 2020* | Italy | 24 | 10 y/o | AA | Hypothyroidism | NR | NR | Fair |
| *Scotson, 1989* | UK | 27, M | NR | AT | Hypothyroidism | Thyroxine (50 ug), improvement after 12 months | NR | Fair |
| *Schepis, 2017* | Italy | 30, M | 15 y/o | AA | Hypothyroidism | NR | NR | Poor |

**Abbreviations**: AA – alopecia areata; AT – alopecia totalis; AU – alopecia universalis; b.i.d. – twice daily; CR – complete resolution; DNCB – dinitrochlorobenzene; FAPG - fatty alcohol propylene glycol; NR – not reported; PUVA – psoralen and ultraviolet A radiation; q.d. – once daily; ROB – risk of bias assessment; y/o – year(s) old
