# Supplementary material for: Common Dermatologic Disorders in Down Syndrome: Systematic Review
Source: JMIR Dermatol. 2022 Feb 8;5(1):e33391. doi: 10.2196/33391 (PMC10334906; doi:10.2196/33391)
Supplement: Multimedia Appendix 6 [file derma_v5i1e33391_app6.docx]

# Summary of case reports of Down syndrome patients with scabies infestation

| **Study** | **Country** | **Type of infestation** | **Age, Sex** | **Affected areas** | **Other skin conditions** | **Effective treatment** | **Failed or previous treatments** | **ROB** |
| --- | --- | --- | --- | --- | --- | --- | --- | --- |
| *Fonseca, 2014* | USA | Scabies | 3, F | Scalp, neck, elbows, hands, feet | NR | Oral ivermectin (200 ug/kg once weekly for 4 weeks) and 2 courses of permethrin 5% cream 1 week apart; significant improvement of hyperkeratosis and pruritus, but persistent onychodystrophy | Oral antifungal for presumptive onychomycosis; Triamcinolone 0.1% ointment for suspected psoriasis; Cyclosporine 5 mg/kg/day with worsening hyperkeratosis after 5 weeks | Fair |
| *Assaf, 2016* | USA | Scabies (Norwegian or crusted) | 8, F | Whole body, mostly on hands and feet | NR | Single oral dose (200 ug/kg) of ivermectin; permethrin 5% topical cream; with CR in 3 days | Treated with 1 month of hydrating cream, 4 months of triamcinolone cream for presumed eczema | Fair |
| *Lee, 2019* | USA | Scabies | 11, F | Scalp; extremities | Severe AD (treated with topical triamcinolone 1%) | 7 daily doses of oral ivermectin (200 mg/kg), topical permethrin 5% for 2 weeks, and clindamycin, with improvement in 1 week | NR | Fair |
| *Senterre, 2020* | Belgium | Scabies | 26, F | Face, extremities | NR | Oral ivermectin (200 ug/kg) and permethrin cream 30 g daily, with CR in 1 month | Clobetasol propionate 0.05% ointment; Betamethasone and calcipotriol ointment; UVB-phototherapy; oral acitretin; Risankizumab | Good |
| *Tschen, 1980* | USA | Scabies | 41, F | NR | EPS | NR | Iodine, ethyl chloride, liquid nitrogen, tretinoin, topical steroids, tar, vitamins A and E, griseofulvin, zinc sulfate, methotrexate, salicylic acid, and sulfur | Poor |
| *Nagsuk, 2015* | USA | Scabies | 46, F | Hands, feet, arms, legs, neck, face | Bacterial superinfection with E coli, staph aureus, and streptococcus agalactiae | 2 doses of oral ivermectin 200 ug/kg and 2 doses of topical 5% permethrin, with CR in 2 months | Treated with triamcinolone, clobetasol, ciclopirox, nystatin, terbinafine and fluconazole for presumed eczema and tinea corporis | Good |
| *Jayananda, 2013* | USA | Scabies (Norwegian or crusted) | 67, F | Hands, feet, neck | Hospitalized for secondary bacterial infection of skin with septicemia | Oral ivermectin and topical 5% permethrin cream | Permethrin cream and prednisone taper (improvement in skin findings); topical steroids and oral methotrexate with initial improvement, but then worsening of dermatitis and keratoderma and adverse effects from methotrexate | Fair |

**Abbreviations:** CR – complete resolution; NR – not reported; ROB – risk of bias assessment
