# Supplementary material for: Common Dermatologic Disorders in Down Syndrome: Systematic Review
Source: JMIR Dermatol. 2022 Feb 8;5(1):e33391. doi: 10.2196/33391 (PMC10334906; doi:10.2196/33391)
Supplement: Multimedia Appendix 7 [file derma_v5i1e33391_app7.docx]

# Summary of case reports of Down syndrome patients with leishmaniasis infestation

| **Study** | **Country** | **Type of infestation** | **Age, Sex** | **Affected areas** | **Comorbidities** | **Effective treatment** | **Failed or previous treatments** | **ROB** |
| --- | --- | --- | --- | --- | --- | --- | --- | --- |
| *Abass, 2009* | Saudi Arabia | Leishmaniasis | 2 months, M | Face, 3 ulcers | NR | NR | Sodium antimony gluconate IV with improvement, but discharged after 2 weeks at insistence of parents | Good |
| *Villibor, 2019* | Brazil | Leishmaniasis | 20 months, F | Upper lip | NR | 1 mg/kg/day of amphotericin B, ceftriaxone (100 mg/kg/day), oxacillin (200 mg/kg/day), and methylprednisolone (2 mg/kg/day), CR in 15 days, no recurrence at 8 months | NR | Fair |
| *Aghaei, 2004* | Iran | Leishmaniasis (*Leishmania major*) | 35, M | Face, trunk, extremities | Hypothyroidism, T2DM, vitiligo | IM injections of meglumine antimoniate (Glucantime, 1 vial/day) with oral ketoconazole (200 mg/q 8h) for 20 days | IM injections of meglumine antimoniate (Glucantime, 3 vials/day), but patient became leukopenic after 7 days | Good |
| *Ferreli, 2004* | Italy | Leishmaniasis (*Leishmania infantum*) | 57, F | Inferior lip | Cataracts; recurrent gingivitis, multiple dental caries | Intralesional meglumine antimoniate (Glucantim) 1 mL once weekly with CR in 1 month | Topical and general antibiotics, anti-inflammatory and antihistaminic medication, corticosteroid creams; intramuscular meglumine antimoniate (Glucantim) 60 mg/kg/day; after 15 days of treatment, occurrence of 2nd degree AV heart block required discontinuation of medication | Fair |

**Abbreviations:** CR – complete resolution; NR – not reported; ROB – risk of bias assessment
