# Supplementary material for: Common Dermatologic Disorders in Down Syndrome: Systematic Review
Source: JMIR Dermatol. 2022 Feb 8;5(1):e33391. doi: 10.2196/33391 (PMC10334906; doi:10.2196/33391)
Supplement: Multimedia Appendix 8 [file derma_v5i1e33391_app8.docx]

# Summary of case reports of Down syndrome patients with calcinosis cutis

| **Study** | **Country** | **Age, Sex** | **Age of onset** | **Affected areas** | **Presence of syringomas** | **Treatment or resolution** | **ROB** |
| --- | --- | --- | --- | --- | --- | --- | --- |
| *Turan, 2016* | Turkey | 4, F | 8 months prior | Dorsa of hands, wrists | Palpebral | NR | Fair |
| *Kumar, 2016* | India | 5, F | 1.5 y/o | Face, dorsa of hands, neck, extremities | NR | Topical sodium thiosulfate compounded in zinc oxide applied to existing lesions | Good |
| *Kanzaki, 1991* | Japan | 6, F | 1 year prior | Hands, wrists, feet | Palpebral and perilesional | NR | Fair |
| *Smith, 1989* | USA | 6, M | 2 years prior | Hands, wrists, elbows, knees, face | None | NR | Fair |
| *Solak, 2016* | Turkey | 6, F | 3 years prior | Hands, feet, face | NR | NR | Fair |
| *Kotsuji, 2001* | Japan | 7, F | 2 years prior | Fingers, palms, dorsum of hands | None | NR | Fair |
| *Sais, 1995* | Spain | 7, F | 1 year prior | Hands, feet | NR | Some spontaneous resolution | Fair |
| *Fox, 2013* | USA | 8, F | Several years prior | Dorsa of hands | NR | NR | Fair |
| *Schepis, 1996* | Italy | 10, M | NR | Palms, fingers, soles | Perilesional | NR | Fair |
|  |  | 11, M | NR | Dorsum of Lt hand, toes | None | NR |  |
| *Lucky, 2002* | USA | 11, F | 4 y/o | Hands, feet, periorbital areas | None | Many lesions spontaneously resolved without scarring | Fair |
| *Motegi, 2019* | Japan | 11, F | 3 y/o | Face, neck, hands, knees | Palpebral | NR | Fair |
| *Schepis, 1994* | Italy | 11, F | Several years prior | Hands, feet | Palpebral | NR | Fair |
| *Maroon, 1990* | USA | 12, M | 2 years prior | Hands and forearms, later on thighs, face, neck | Perilesional and palpebral | NR | Fair |
| *Hattori, 2018* | Japan | 14, M | 6 months prior | Bilateral auricles | NR | NR | Fair |

**Abbreviations**: NR – not reported; ROB – risk of bias assessment
