# Supplementary material for: Common Dermatologic Disorders in Down Syndrome: Systematic Review
Source: JMIR Dermatol. 2022 Feb 8;5(1):e33391. doi: 10.2196/33391 (PMC10334906; doi:10.2196/33391)
Supplement: Multimedia Appendix 9 [file derma_v5i1e33391_app9.docx]

# Summary of case reports of Down syndrome patients with dermatofibromas

| **Study** | **Country** | **Age, Sex** | **Age of onset** | **No. of lesions** | **Affected areas** | **Medical history** | **ROB** |
| --- | --- | --- | --- | --- | --- | --- | --- |
| *Tanaka, 2017* | Japan | 15, F | 6 months prior | 6 | Right thigh, right shoulder, brachium and hip following Blaschko’s lines | No evidence of autoimmune disease, immunodeficiency or HIV infection | Fair |
| *Honda, 2016* | Japan | 26, F | 19 | 7 | Trunk and limbs bilaterally | History of acute megakaryoblastic leukemia at 3 months of age (treated with chemotherapy), but not in obvious immunosuppressive state at time of onset | Fair |
| *Lamb, 2014* | UK | 65, M | Several months prior | 20-30 | Upper arms, lower legs bilaterally | Mild lymphopenia but otherwise no evidence of immunosuppression; no evidence of autoimmune conditions | Fair |

**Abbreviations:** ROB – risk of bias assessment
