# Supplementary material for: Common Dermatologic Disorders in Down Syndrome: Systematic Review
Source: JMIR Dermatol. 2022 Feb 8;5(1):e33391. doi: 10.2196/33391 (PMC10334906; doi:10.2196/33391)
Supplement: Multimedia Appendix 10 [file derma_v5i1e33391_app10.docx]

# Summary of cases of Down syndrome patients with confirmed melanoma

| **Study** | **Country** | **Age, Sex** | **Type** | **Affected areas** | **Stage / Clark level** | **Ulceration** | **Metastasis** | **Current treatment** | **ROB** |
| --- | --- | --- | --- | --- | --- | --- | --- | --- | --- |
| *Jafarian, 2005* | Canada | 11, F | NR | Leg | Stage IIA | NR | None | Surgical excision | Fair |
| *Satge, 2014* | France | 19, F | SSM | Lumbar region | Clark level II | No ulceration | None | Surgical therapy | Fair |
| *Nakano, 1993* | Japan | 39, F | ALM | Right foot | Clark level V | Central ulcer | None | Surgical excision | Good |

**Abbreviations:** ALM – acral lentiginous melanoma; NR – not reported; ROB – risk of bias assessment; SSM – superficial spreading melanoma
