# Supplementary material for: Common Dermatologic Disorders in Down Syndrome: Systematic Review
Source: JMIR Dermatol. 2022 Feb 8;5(1):e33391. doi: 10.2196/33391 (PMC10334906; doi:10.2196/33391)
Supplement: Multimedia Appendix 11 [file derma_v5i1e33391_app11.docx]

# Summary of case reports of Down syndrome patients with syringoma(s)

| **Study** | **Country** | **Age, Sex** | **Affected areas** | **Other skin conditions** | **ROB** |
| --- | --- | --- | --- | --- | --- |
| *Turan, 2016* | Turkey | 4, F | Left upper eyelid | MICC | Fair |
| *Kanzaki, 1991* | Japan | 6, F | Upper and lower eyelids | MICC | Fair |
| *Motegi, 2019* | Japan | 11, F | Jaw | MICC | Fair |
| *Schepis, 1994* | Italy | 11, F | Lower eyelids | MICC | Fair |
| *Maroon, 1990* | USA | 12, M | Upper and lower eyelids | Calcinosis cutis | Fair |
| *Seo, 2007* | Korea | 13, F | Periocular, perioral, both axillae | NR | Fair |
| *Ong, 2010* | Singapore | 18, F | Eruptive syringoma over trunk and limbs, over 1 month | NR | Fair |
| *Togawa, 2003* | Japan | 31, F | Forearm | Collagenoma | Fair |

**Abbreviations:** MICC – milia-like idiopathic calcinosis cutis; NR – not reported; ROB – risk of bias assessment
