# Supplementary material for: Common Dermatologic Disorders in Down Syndrome: Systematic Review
Source: JMIR Dermatol. 2022 Feb 8;5(1):e33391. doi: 10.2196/33391 (PMC10334906; doi:10.2196/33391)
Supplement: Multimedia Appendix 12 [file derma_v5i1e33391_app12.docx]

# Summary of case reports of Down syndrome patients with elastosis perforans serpiginosa (EPS)

| **Study** | **Country** | **Age, Sex** | **Affected areas** | **Comorbidities** | **Effective treatment** | **Failed or previous treatments** | **ROB** |
| --- | --- | --- | --- | --- | --- | --- | --- |
| *Rasmussen, 1972* | USA | 12, M | Neck, arms, legs | NR | NR | NR | Fair |
|  |  | 13, M | Neck, arms, legs | NR | Spontaneous resolution in 3 years | NR |  |
|  |  | 14, F | Neck, arms, legs | NR | NR | NR |  |
|  |  | 16, M | Neck, arms, legs | NR | NR | NR |  |
| *Abudullah, 2010* | USA | 16, M | Arms | NR | Tazarotene gel 0.1% q.d., complete resolution in 2 months | NR | Fair |
| *Espinosa, 2008* | USA | 16, F | Arms, legs | CVA, moyamoya disease | NR | NR | Good |
| *Hernandez-Ruiz, 2015* | Spain | 16, M | Legs | NR | NR | Topical betamethasone and calcipotriol | Good |
| *Polanska, 2016* | Poland | 16, F | left cheek; arms and legs | Hypothyroidism, tetralogy of Fallot and bilateral congenital dislocation of the hip joints; psoriasis | NR | NR | Good |
| *Siragusa, 1997* | Italy | 16, M | Left mandibular angle | NR | Spontaneous resolution in 6 months with no recurrence 12 months | NR | Good |
| *Mehta, 2001* | UK | 16, M | Elbows, neck | osteogenesis imperfecta | NR | Haelan tape, Retin-A, cryotherapy, curettage, and Dovonex ointment | Good |
|  |  | 21, M | Beginning on face, spreading to trunk | NR | Partial improvement with curettage | Topical corticosteroids |  |
|  |  | 22, F | Bilateral neck and upper arms | EDS type IV | Neck lesions spontaneously resolved after several years | Topical steroids, 0.25%; Retin-A gel; 2% salicylic acid ointment; oral isotretinoin (25 mg) q.d.; intralesional corticosteroid and repeated cryotherapy, curettage and cautery, trial of nbUVB, phenytoin |  |
| *Crotty, 1983* | USA | 19, M | Neck, forearms, thighs, calves | NR | NR | NR | Fair |
| *Pereira, 2010* | Brazil | 19, F | Right arm and knee | Hypercholesterolemia, (simvastatin 20 mg/day) | Partial remission with cryotherapy with LN | NR | Good |
| *De Pasquale, 2002* | Italy | 20, F | Thighs | Hypothyroidism (oral levothyroxine 50 ug/day); acne, hypertrichosis, onychomycosis, cheilitis | NR | NR | Fair |
| *Scherbenske, 1990* | USA | 24, F | Arms and legs | NR | NR | NR | Good |
| *Treadwell, 1990* | USA | 25, F | Extensor forearms and knees | NR | NR | Flurandrenolide cream 0.05%; Flurandrenolide tape | Good |
| *Kaufman, 2000* | USA | 26, M | Arms, legs | NR | Flashlamp pulsed dye laser; no recurrence at 14 months | Tretinoin and glycolic acid | Fair |
| *Kaufman, 1975* | USA | 27, F | Arms | Psoriasis, acanthosis nigricans | NR | NR | Fair |
| *O’Donnell, 1992* | Ireland | 28, F | Extensor surfaces of arms and legs | Cholelithiasis; hepatic dysfunction | Mild improvement with topical moisturizing agents | NR | Fair |
| *Suneja, 2007* | USA | 41, M | Forearms | NR | NR | NR | Fair |
| *Tschen, 1980* | USA | 41, F | NR | Scabies (all mites found in EPS lesions) | NR | Topical agents (Iodine, ethyl chloride, LN, tretinoin, topical steroid preparations, tar, MTX, salicylic acid, sulfur); Systemic treatments (griseofulvin, zinc sulfate, steroids) | Poor |
| *Gregersen, 2010* | Denmark | 45, M | Forearms, thighs | NR | Topical therapy with imiquimod 5% cream | Topical corticosteroids, systemic antifungals | Fair |

**Abbreviations:** CVA – cerebrovascular accident; EDS – Ehlers-Danlos syndrome; EPS – elastosis perforans serpiginosa; LN – liquid nitrogen; MTX – methotrexate; nbUVB – narrow band ultraviolet B; NR – not reported; q.d. – once daily; ROB – risk of bias assessment
